# *In Vitro* Biofilm Formation and Antibiotic Susceptibility Patterns of Bacteria from Suspected External Eye Infected Patients Attending Ophthalmology Clinic, Southwest Ethiopia

**DOI:** 10.1155/2020/8472395

**Published:** 2020-03-18

**Authors:** Kuma Diriba, Tesfaye Kassa, Yared Alemu, Sisay Bekele

**Affiliations:** ^1^Department of Medical Laboratory Sciences, Health Science and Medical College, PO Box 419, Dilla University, Dilla, Ethiopia; ^2^School of Medical Laboratory Science, PC-Bldg, PO Box 788, Jimma University, Jimma, Ethiopia; ^3^School of Medical Laboratory Science, PO Box 378, Jimma University, Jimma, Ethiopia; ^4^Department of Ophthalmology, Jimma University, Jimma, Ethiopia

## Abstract

**Background:**

Ocular disease with its complications is a major public health problem which has significant impacts on the quality of life particularly in developing countries. An eye infection due to bacterial agents can lead to reduced vision and blindness. This study was aimed to assess the antimicrobial susceptibility pattern and biofilm-forming potential of bacteria isolated from suspected external eye infected patients in Jimma.

**Method:**

A cross-sectional facility-based study was conducted on 319 suspect patients with external eye infections from March to June 2017 at Jimma University Medical Center (JUMC) Ophthalmology Department in Ethiopia. External ocular specimens were collected and standard operating procedures were followed to handle and culture throughout the study period. Antimicrobial susceptibility was determined by the disk diffusion method according to CLSI guidelines. Microtiter (96 wells) plate method was used to screen biofilm formation by ELISA reader at 570 nm.

**Results:**

Out of 319 study participants with an external eye infection, the prevalence of bacterial pathogens was 46.1%. The predominant bacterial isolates were coagulase-negative staphylococcus (CoNS) (27.7%) followed by *Staphylococcus aureus* (19.7%). Among Gram-negative groups, *Pseudomonas aeruginosa* (6.8%) was the leading isolate. Increased antimicrobial resistance was observed for tetracycline (64%), erythromycin (66.7%), and penicillin (77.1%). Amoxicillin-clavulanic acid, ciprofloxacin, and gentamicin were the most effective drugs for external eye infections due to susceptibility ranging from 70 to 100% among both Gram-negative and Gram-positive groups. Methicillin-resistant *S. aureus* (MRSA) accounted for 13.8%. Multidrug resistance (MDR) accounted for 68.7%. The overall biofilm formation rate of bacterial ocular pathogens was 66.1%, where *P. aeruginosa* (40%), CoNS (34.1%), and *S. aureus* (31%) formed strong biofilm phenotype.

**Conclusion:**

The prevalence rate of bacterial isolates was high. Almost all bacterial isolates were resistant to at least one or more drugs. MDR pathogens were observed increasingly among biofilm formers or vice versa.

## 1. Introduction

The human eye, which is constantly exposed to the external environment, is a unique organ serving as the window of our body. Ocular disease with its complications, due to microorganisms, is a significant health problem worldwide particularly in the least income countries [1]. Ocular infections can damage the structure of the eye which can lead to reduced vision or even blindness if it is inappropriately diagnosed and treated. The most frequently affected parts of the eye due to microorganisms are the conjunctiva, eyelid, and cornea [1]. Conjunctivitis, blepharitis, and dacryocystitis are considered the most common manifestations of external eye infections [2]. These pathogenic microorganisms include bacteria, fungi, viruses, and parasites [3].

Bacteria are the major causative agents of external eye infections in Jimma area [4]. Frequently, control of eye infections may involve the use of broad-spectrum antimicrobial agents. Nevertheless, the emerging and increasing antimicrobial resistance is a problem worldwide [5]. In this regard, inappropriate and irrational use of antimicrobial medicines provides favorable conditions for resistant microbes to emerge, spread, and persist [6]. The development of bacterial biofilms is presently recognized as one of the most relevant drivers of persistent infections. Bacterial biofilm formation constitutes a serious challenge for clinical microbiologists and physicians being 100- to 1000-fold more resistant to antimicrobial agents than their counterparts in planktonic forms [7]. Phenotypic and physiological changes in the biofilm platform restrict the penetration of antibiotics into biofilm-forming bacteria and as a result provide a higher resistance to antimicrobial treatments [8].

In most parts of Ethiopia, antibiotics without prescription are available free of trouble. This can lead to overuse or misuse of antibiotics [9] and can, in turn, contribute to the emergence and spread of antimicrobial-resistant strains. Moreover, in developing countries, sanitary practices in the facial area are poor that may play a part in the increased prevalence of bacterial eye infections. The rising antimicrobial resistance increases the risk of treatment failure with potentially serious consequences [10, 11].

Even though a study on ocular infection was conducted in 2012 in Jimma area [4], the bacterial profile and antimicrobial susceptibility pattern can vary from time to time and place to place as indicated in different studies [3, 12]. Therefore, the changing spectrum of microorganisms involved in external eye infections and the emergence of acquired microbial resistance to antibiotics need continuous surveillance to guide empirical therapy. As a result of this, updated knowledge of bacterial etiologic agents in eye infections and their antibiogram are crucial. On the other hand, bacterial biofilm production was not addressed in isolates from external ocular infections in Jimma area as well as in Ethiopia. Hence, the present study was intended to update the bacterial profile present in external eye infections and their antimicrobial susceptibility pattern along with biofilm-forming potential of the isolates at Jimma University Medical Center (JUMC) Ophthalmic clinic, Southwest Ethiopia.

## 2. Materials and Methods

### 2.1. Study Design and Population

A cross-sectional health-facility-based study was conducted on a total of 319 patients consecutively attending Ophthalmology Clinic at JUMC from March to June 2017. All patients with external eye infections that fulfill the eligibility criteria during the study period were recruited prospectively by an ophthalmic nurse and confirmed based on clinical examination by ophthalmologists. Patients examined and diagnosed with a slit lamp (above 4 years of age) and who had an external ocular infection with red-eye, discharge, mucoid, or mucopurulent secretion, had thickening of the conjunctiva in one or both eyes, and agreed to participate were included in this study. Patients on antibiotics within the last 5 days prior to sample collection date were excluded from the study since bacteria are less frequently detected in culture-based tests collected after antibiotic use [13]. The Helsinki declaration on the ethical principles for medical research was followed by ethical clearance obtained from Jimma University Ethics Review Board. Informed and written consent and assent was obtained from the study participants before data collection.

### 2.2. Data Collection Procedures and Process

#### 2.2.1. Sociodemographic and Clinical Characteristics

Sociodemographic data (age, sex, monthly income, educational level, occupation, and address), clinical data (history of repeated infections, duration of stay in the hospital, use of contact lenses, surgery, previous antibacterial therapy, systemic diseases, and use of traditional medicine), and others like source of light and use of firewood at home were collected by an ophthalmic nurse from each study participant using a structured questionnaire. Patients with clinical pictures for external ocular infections were diagnosed by an ophthalmologist.

#### 2.2.2. Specimen Collection, Handling, and Transport

All consecutive patients examined clinically were set apart for suspected bacterial infection. Specimens from external eye structures were collected. Briefly, the patient was requested to look up while lowering the eyelid down and the sample was collected from one or both eyes based on the nature of the infection. Sterile cotton swab that had been premoistened with sterile physiological saline was used gently to collect eye discharge. The swab was rubbed softly over the lower conjunctival sac from medial to lateral side and back again [14]. Purulent material was collected in the cases of dacryocystitis by everted puncta, then applying pressure over the lacrimal sac area from the infected eye [12, 14]. In the cases of ulcerative blepharitis, lashes deposit, tear film foaming content, and corneal punctuate erosions were swabbed. From each patient, two swabs were collected: one for Gram staining immediately after collection and the second for culture. The swab for culture was inserted into Amies transport media with charcoal (Himedia®, India), placed in a cold box, and transported to Jimma University Medical Microbiology Laboratory. Standard operating procedures were followed in handling eye specimens collected throughout the study period [14].

#### 2.2.3. Isolation and Identification of Bacterial Pathogens

The specimens were streaked onto MacConkey agar, Mannitol salt agar, blood agar, and chocolate agar plates (all media were from Oxoid, Hampshire, UK). The plates were incubated at 37°C aerobically for 24 to 48 hours. Inoculated chocolate agar plate was kept in a 5 to 10% CO_2_ atmosphere at 37°C for 24 to 48 hours. All plates were examined initially after 24 hours and cultures with no growth were further incubated overnight. Bacteria were identified on the basis of phenotypic and a series of biochemical tests. For *Haemophilus* spp., satellitism test was also performed [11].

#### 2.2.4. Bacterial Biofilm Tests

Briefly, bacteria isolated from fresh agar plates were inoculated into a tube filled with sterile tryptone soya broth (TSB) with 1% glucose and incubated at 37°C for 24 hours. This culture was diluted 1 : 100 into the fresh media. Then, 200 *μ*L of the suspension was added to a sterile 96-well flat-bottom microtiter plate and incubated at 37°C for 48 hours. The bacterial suspension of each well was gently spent and washed three times with phosphate buffer saline (pH 7.2). Plates were fixed with absolute methanol and then stained with 220 *μ*L crystal violet (CV, 0.1% w/v) for 15 min at room temperature. Each well was washed three times with PBS to remove unbound CV dye. After drying, 220 *μ*L of ethanol (95%) was added to each well. Finally, the solubilized CV was transferred to a new microtiter plate. The optical density (OD) of the biofilm was measured by a microplate ELISA reader (HumaReader HS, German) at a wavelength of 570 nm. The experiment was performed in triplicate separately for each strain and the average value was calculated [15].

The cut-off optical density (ODc) was proof of the biofilm formation and was defined as the sum of the arithmetic mean of negative controls and a triple value of its standard deviation (ODc = ẍ + 3*σ*). TSB without bacterial suspension incubated in the microtiter plate was used as a negative control. Biofilm formation of the isolates was classified into four categories as stated in a previous study [16]: nonadherent (OD < ODc), weakly adherent (ODc < OD < 2 *∗* ODc), moderately adherent (2 *∗* ODc < OD < 4 *∗* ODc), and strongly adherent (4 *∗* ODc < OD).

#### 2.2.5. Antimicrobial Susceptibility Testing

It was carried out using the Kirby–Bauer disk diffusion method as recommended by the Clinical and Laboratory Standard Institute (CLSI) guideline [17]. From a pure culture, three to five colonies of the test organism were emulsified in 3 ml of sterile normal saline, and the suspension was adjusted to a 0.5 McFarland standard. Fifteen impregnated antibiotic disks were used in the following concentrations: amikacin (30 *μ*g), ampicillin (10 *μ*g), amoxicillin-clavulanic acid (20 *μ*g), cefoxitin (30 *μ*g), ceftazidime (30 *μ*g), ceftriaxone (30 *μ*g), chloramphenicol (30 *μ*g), ciprofloxacin (5 *μ*g), clindamycin (2 *μ*g), erythromycin (15 *μ*g), gentamicin (10 *μ*g), penicillin G (10 IU), tetracycline (30 *μ*g), trimethoprim-sulphamethoxazole (1.25/23.75 *μ*g), and tobramycin (10 *μ*g) (all antibiotics were from Oxoid, Hampshire, UK). These drugs were placed in the lawn plate and incubated at 37°C for 18–24 hours. For fastidious bacteria, 5% fresh or heated sheep blood in Muller Hinton agar base was used. The zone of inhibition was measured and interpreted accordingly. Methicillin-resistant isolates were determined using cefoxitin disk (30 *μ*g) by incubating at 34 ± 1°C as recommended by CLSI [17]. Multidrug resistance (MDR) is operationalized as a resistance of bacterium to at least one agent in three or more different classes of antibiotic drugs [18].

#### 2.2.6. Data Quality Assurance

All external eye specimens were collected following standard operating procedure by ophthalmologist and ophthalmic nurse. Cross-checking was done on a daily basis for an incomplete patient profile. All laboratory and clinical data were recorded during the study period as a backup. The sterility of culture media was ensured by incubating five percent of each batch of the prepared media at 37°C for 24 hours. For better results, any physical changes like cracks, excess moisture or dehydration, color change, hemolysis, contamination, deterioration, and expiration dates were checked before or at the time of using reagents and culture media. The quality and performance of culture media, biochemical tests, and antimicrobial susceptibility discs were checked using *E. coli* (ATCC 25922), *S. aureus* (ATCC 25923), *P. aeruginosa* (ATCC 27853), and *S. pneumoniae* (ATCC 49619), all obtained from Ethiopian Public Health Institute, Addis Ababa.

#### 2.2.7. Data Processing and Analysis

Data entry, analysis, and cleaning were done using Epi-Data 3.1 and SPSS version 21.0 software. Frequency count and percentage were used to present the finding. Prevalence figures were calculated for the total study population and separately by the clinical feature of the disease. Multivariate logistic regression was used to assess the significantly associated variable with bacterial prevalence. Potential associated factors were identified by bivariate analysis with *p* < 0.25 as a candidate for checking in multivariate logistic regression model and *p* < 0.05 was considered statistically significant.

## 3. Results

### 3.1. Sociodemographic and Clinical Features of Study Participants

A total of 319 study participants diagnosed clinically with external ocular infection were included in the study. The age ranged from 1 month to 95 years with a median of 21 years old and 172 (53.9%) were male patients. The majority of study subjects were children below the age of two years which accounts for 103 (32.3%) followed by above 45 years of age groups accounting for 74 (23.2%). Bivariate analysis (COR) did not show a significant association between sociodemographic characteristics and bacterial isolation patterns. A small proportion of study participants had additional chronic diseases like hypertension 18 (5.6%), diabetes 17 (5.3%), and rheumatoid arthritis 11 (3.4%). Twenty-one (6.6%) study participants were previously hospitalized for an eye infection, and 31 (9.7%) had used topical medicine for eye treatment. Five (1.6%) study participants had eye surgery and 23 (7.2%) cases had used traditional eye remedies from their local herbal products. Only 5 (1.6%) study participants had contact lens. In the multivariate analysis, patients with diabetes (AOR = 0.09, 95% CI: 0.02–0.43, *p*=0.002) and previous history of hospitalization (AOR = 0.10, 95% CI: 0.03–0.42, *p*=0.001) were significantly associated with the occurrence of external eye bacterial infections.

On the clinical ground, from all 319 patients, 165 (51.7%) were with conjunctivitis, followed by 74 (23.2%) with blepharoconjunctivitis, 52 (16.3%) with blepharitis, 13 (4.1%) with dacryocystitis, and 15 (4.7%) with other external eye infections. The most dominant external ocular infection was conjunctivitis with a significant pediatric age group affected.

### 3.2. Prevalence of Bacterial Isolate

Out of 319 ocular specimens processed for culture, bacteria were isolated from 147 giving an overall prevalence of 46.1%. No mixed bacterial isolate per patient was found in this study. Among the bacterial isolates, 96 (65.3%) of them were Gram-positive groups and the remaining 51 (34.7%) were Gram-negative groups. From the former groups, CoNS was the most frequent isolate accounting for 41 (27.9%), followed by *S. aureus* and *S. pneumoniae* with 29 (19.7%) and 13 (8.8%), respectively. From the latter groups, *P. aeruginosa* was the predominant isolate accounting for 10 (6.8%), followed by 9 (6.1%) *K. pneumoniae*. The spectrum of bacterial isolate varies with the age of patients. Most of the bacterial isolates were recovered from cases that were between one month and two years of age group (Table 1).

Most of the bacterial isolates were recovered from 75 (51.0%) conjunctivitis cases followed by 32 (21.8%) blepharitis and 27 (18.4%) blepharoconjunctivitis cases. The least bacterial isolates were found in 8 (5.4%) dacryocystitis cases. The predominant isolates among conjunctivitis cases were CoNS which accounted for 16 (21.3%) followed by 15 (20%) *S. aureus.* In blepharitis, the leading bacterial etiologies have a similar pattern with conjunctivitis cases: 10 (31.2%) CoNS and 8 (25%) *S. aureus*. In blepharoconjunctivitis, 10 (37%) CoNS followed by 3 (11.1%) *H. influenzae* were identified, whereas in dacryocystitis, 3 (37.5%) CoNS followed by 2 (25%) *S. pneumoniae* and 2 (25%) *S. aureus* were identified. Among Gram-negative groups, *P. aeruginosa* and *K. pneumoniae* were the predominant isolates among conjunctivitis cases with 5 (6.7%) and 4 (5.3%), respectively. The remaining Gram-positive and negative groups are shown in Table 2.

### 3.3. Antimicrobial Susceptibility Pattern of Bacterial Isolates

In Gram-positive bacteria, twelve antibiotics belonging to nine categories were used. *S. aureus* showed susceptibility to ciprofloxacin for 26 (89.7%) followed by clindamycin, amoxicillin-clavulanic acid, and gentamicin each accounting for 24 (82.8%), 22 (75.9), and 21 (72.4%), respectively. On the other hand, this bacterium was exceedingly resistant to penicillin, 25 (86.2%); erythromycin, 24 (82.8%); and tetracycline, 22 (75.9%). CoNS showed almost comparable susceptibilities as that of *S. aureus* for the above antimicrobials. Among *S. aureus* isolates, 4 (13.8%) of them were MRSA phenotype. From CoNS isolates, 12 (29.3%) of them were also methicillin-resistant. *S. pneumoniae* showed 100% susceptibility to amoxicillin-clavulanic acid whereas 11 (84.6%) to ciprofloxacin. *S. pneumoniae* was more resistant to penicillin and trimethoprim-sulphamethoxazole; each equally accounts for 9 (69.2%). Other Gram-positive groups were susceptible to amoxicillin-clavulanic acid (100%), clindamycin (93.3%), and gentamicin (86.7%) while they were nonsusceptible proportionally to trimethoprim-sulphamethoxazole, tetracycline, and ampicillin (Table 3).

Among Gram-negative groups, 9 (90%) of *P. aeruginosa* isolates were susceptible to ciprofloxacin, followed by ceftriaxone and amoxicillin-clavulanic acid, each accounting for 8 (80%), and gentamicin and amikacin, each accounting for 7 (70%). Contrarily, *P. aeruginosa* isolates showed resistance to ceftazidime for 9 (90%), tetracycline for 8 (80%), and trimethoprim-sulphamethoxazole and tobramycin each accounting for 7 (70%). All *K. pneumoniae* isolates showed susceptibility to ceftriaxone or ciprofloxacin followed by 8 (88.9%) of the isolates which were susceptible to each of amoxicillin-clavulanic acid, chloramphenicol, and amikacin. However, this bacterium was not susceptible to tobramycin, trimethoprim-sulphamethoxazole, and tetracycline. The results of other Gram-negative groups tested show susceptibility or resistance as depicted in Table 4.

MDR was recorded in 101 (68.7%) of 147 total bacterial isolates. From Gram-positive and Gram-negative groups, 78.1% (75/96) and 51.0% (26/51) were MDR, respectively. Among Gram-positive groups, a high level of MDR was found in 25 (86.2%) *S. aureus* followed by 34 (82.9%) CoNS. Among Gram-negative groups, all (*n* = 10) *P. aeruginosa* strains showed MDR followed by 6 (66.7%) *K. pneumoniae* and 3 (60.0%) *E. coli* (Table 5).

### 3.4. Biofilm Formation Profile

**Figure 1:**
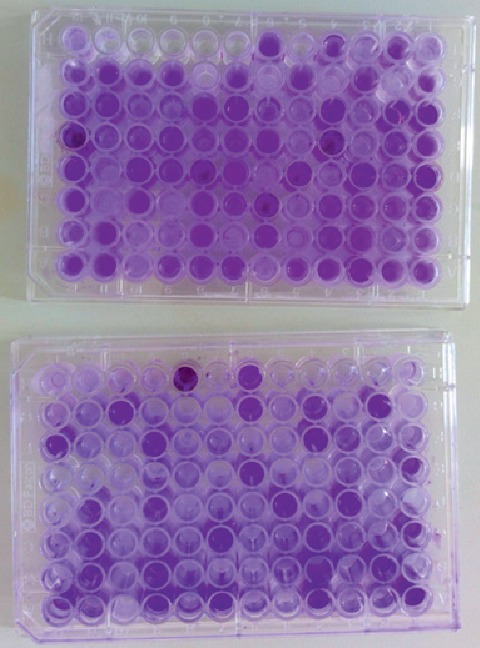
Microtiter polystyrene (96-well) plate method used to categorize bacterial isolates as strong, moderate, weak, and nonbiofilm producers differentiated by crystal violet stain at JUMC.

From 127 bacterial isolates screened for biofilm formation, 84 (66.1%) of them were capable of biofilm production. The strength of bacterial biofilm production was categorized into four groups as derived from the microtiter plate OD reading (Figure 1), i.e., 31 (24.4%) of them as strong former, 39 (30.7%) as moderate, 14 (11.0%) as weak, and 43 (33.9%) as nonbiofilm former. Among 83 Gram-positive isolates, 56 (67.5%) of them were biofilm formers. Strong biofilm formers were seen in 14 (34.1%) CoNS followed by 9 (31.0%) *S. aureus*. Generally, about 76% and 72% of each of CoNS and *S. aureus* isolates were biofilm former, respectively. On the other hand, from the Gram-negative groups, 4 (40.0%) *P. aeruginosa* were strong biofilm former followed by 2 (22.2%) *K. pneumoniae* (Table 6).

### 3.5. Correlation of Antimicrobial Resistance and Biofilm Formation

**Figure 2:**
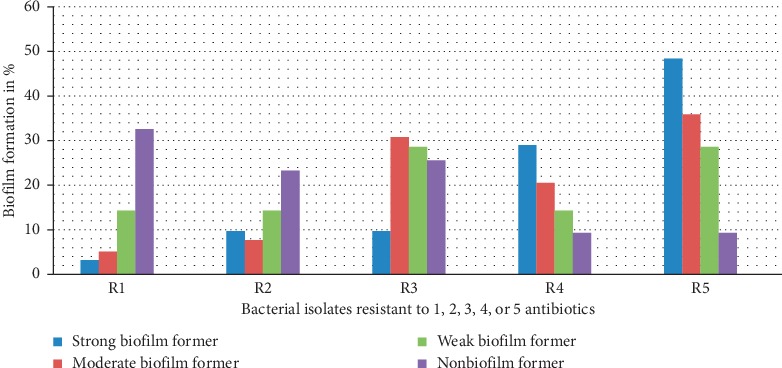
Relationship of antimicrobial resistance and biofilm formation of isolates from JUMC eye clinic.

Nonbiofilm formers predominated among those bacterial isolates with resistance to one or two antimicrobial agents. But in bacterial isolates where there were MDR features (resistance to three or more drugs in different classes of antimicrobials), higher numbers of biofilm formers were the principal ones. Significant rates of strong biofilm formers (48.4%) were seen in bacterial isolates that were resistant to five or more antimicrobial agents. In this study, the Chi-square statistic revealed a significant relationship between MDR and biofilm former bacterial isolates with *p* < 0.05, despite the fact that the correlation coefficient (0.491) did not show strength (Figure 2).

## 4. Discussion

A total of 319 patients suffering from external ocular infections were included in four months period in 2017. The overall prevalence of bacterial external eye infection rate was 46.1%. This result is comparable with a previous study conducted in Southern Ethiopia [18]. However, the result is lower than the prevalence reported from elsewhere ranging between 74% and 88% [4, 19, 20]. But our study finding is higher than the study conducted in Bangalore [11]. The different rate of bacterial isolation from one place to another or within the same place might be due to different factors including geographic variation, lack of awareness for proper sanitary measures, specimen collection and transportation methods, media used for culturing bacteria, differences in study period, and case inclusion criteria. This study only attempted to isolate aerobically cultivable bacteria without looking into anaerobic bacteria, fungal, and *Chlamydia trachomatis* etiologies.

On a clinical diagnosis process, conjunctivitis was the predominant external ocular infection accounting for 51.7% followed by blepharoconjunctivitis (23.2%), blepharitis (16.3%), dacryocystitis (4.1%), and other eye infection accounting for the remaining 4.7%. This is similar to previous studies conducted in Ethiopia [18] and India [19], where conjunctivitis is frequently the leading causes of external ocular infections. In contrast to this study; however, one finding in southwest Ethiopia [4] reported blepharoconjunctivitis as the predominant type of external ocular infection. The differences within the study might be due to differences in the study period, smaller sample size, and varied inclusion criteria of cases.

The most common isolates observed from external eye infected patients were Gram-positive cocci (65.3%). The finding is indicative of Gram-positive cocci as the primary cause of external ocular infections in Jimma area and it is comparable with other previous studies [9, 21, 22]. From the Gram-positive groups, CoNS (27.9%) was the most predominant isolates followed by *S. aureus* (19.7%) and *S. pneumoniae* (8.8%). Similar studies conducted in Ethiopia [23, 24] and India [25] showed a comparable pattern of isolation. The increased predominance of CoNS and *S. aureus* in external ocular infections indicates that these are responsible for a variety of anterior and posterior segments of eye infections emerging probably from the surface of the skin. Over the past 15 years, there has been an increasing documentation of ocular infections due to CoNS [26]. These bacteria are a known nosocomial pathogen and the cause of health care related infections that can extend to the inner surfaces of the eye, partly as a result of the increasing use of medical devices [27]. As a result, CoNS may become the most common cause of postoperative eye infection in recent years [28–31] and thoughtfulness about this bacterium may be required at JUMC eye health settings where there is inpatient service.

The prevalence of Gram-negative bacterial isolate was 34.7%, with *P. aeruginosa* (6.8%) as the leading agent followed by *K. pneumoniae* (6.1%) in this study. Similar studies in different geographic locations [4, 32–34] also reported that *P. aeruginosa* is the most frequent isolate. Transient contamination of patient hand may be the source of infection of external eye structures. Proper sanitary and hygienic measures including frequent hand and face washing with antimicrobial or nonantimicrobial soap can minimize facial area colonizing bacteria which in turn reduce transient organisms like *P. aeruginosa*, *K. pneumoniae*, and *E coli.*

Among the clinical features of external ocular infections, the predominant bacterial isolates identified among cases of bacterial conjunctivitis and blepharitis were CoNS followed by *S. aureus*. In the case of dacryocystitis, in addition to CoNS and *S aureus*, *S. pneumoniae* was also the predominant strains recovered. This is in agreement with several studies conducted in Ethiopia and elsewhere [18, 20–22, 24, 35–40]. Hence, most external eye infected cases may be managed by considering these members of bacterial etiologies.

This study showed an increased rate of antimicrobial resistance to different agents in both Gram-positive and Gram-negative bacteria, which is consistent with findings from Ethiopia [4, 23] and Uganda [36]. In the *in vitro* antimicrobial susceptibility testing, high frequency of resistance to ampicillin, penicillin, erythromycin, trimethoprim-sulphamethoxazole, tobramycin, and tetracycline has been observed. From these antimicrobial categories, usable medicinal preparations in ophthalmic purpose include tetracycline and tobramycin which needs particular attention at the study place. The observed resistance of the bacteria might be due to the accessibility of most antimicrobial drugs over the counter in Jimma and in Ethiopia at large. In addition, indiscriminate use of antimicrobial drugs and empirical treatment without susceptibility testing results for severe external eye infections by the health professionals, and shortage of routine microbiological services for susceptibility testing and unavailability of updated guideline regarding the selection of drugs are some of the factors which can lead to the development of the increased resistance rate.

In this study, the overall resistance rate of *Staphylococcus* spp. to commonly prescribed antibiotics such as ampicillin, tetracycline, erythromycin, and penicillin was between 73% and 85%. Among these antimicrobials, tetracycline preparation is available in external ophthalmic treatments which may warrant prudent use of it as most *S. aureus* and CoNS were resistant. Consistent results were reported from different studies elsewhere [4, 9, 18, 39]. *P. aeruginosa*, on the other hand, was resistant to ceftazidime (90%) and tetracycline (80%). This is consistent with a study done in Ethiopia where significant proportions of *P. aeruginosa* were resistant to tetracycline [4]. Other Gram-negative isolates including *P. aeruginosa* were resistant to tetracycline and tobramycin in the range between 40% and 80%. Epidemiological factors, study period, and geographic location may be among the factors contributing to highly variable resistance rates. The necessity of bacterial culture and susceptibility for suspected cases of external eye infected patients may be compulsory to select the most effective ophthalmic antimicrobial preparations.

All Gram-positive bacterial isolates were susceptible to amoxicillin-clavulanic acid, ciprofloxacin, and gentamicin within the range of 69%–100%. This is comparable with previous studies done in Ethiopia [18, 23, 40] and India [41]. On the other hand, most Gram-negative bacteria from our study were susceptible to amoxicillin-clavulanic acid, ciprofloxacin, ceftriaxone, and gentamicin within the range of 66%–100%. This is in agreement with previous studies done in different locations [18, 23].

From all *S. aureus* strain isolated, 13.8% were MRSA strains. The finding is comparable with another study reported in India [37]. However, in this study, a significant proportion of CoNS isolates was also methicillin-resistant strains. The most frequent eye infections resistant to methicillin were found among conjunctivitis cases. In this study, those MRSA isolates were susceptible to chloramphenicol (75%) but resistant to clindamycin, tetracycline, and gentamicin. Other similar studies reported chloramphenicol as clinically effective in MRSA (>81%) isolates among conjunctivitis cases and resistant to clindamycin, tetracycline, and gentamicin [42, 43].

About two-thirds of bacterial isolates in this study showed the ability to produce biofilm ranged from weak to strong adherence abilities. This feature can contribute to antimicrobial drug resistance development and play a vital role in pathologic processes over a long period by withstanding the effect of immune defense mechanisms from within and other antimicrobial agents from external eye structures. The bacterial biofilm formation feature is little noticed and studied in Ethiopia. The biofilm formation rate found in this study is comparable with a study conducted in Chicago [44]. However, the comparatively lower biofilm formation rate was reported in Saudi Arabia [45]. *P. aeruginosa* (80%), *K. pneumoniae* (77.8%), CoNS (75.6%), *S. aureus* (72.4%), and *E. coli* (60%) were among the leading biofilm formers. These biofilm-producing features may be responsible for many recalcitrant or refractory infections due to Gram-negative bacilli and Gram-positive cocci and are notoriously difficult to eradicate. It is a well-known pathogenic mechanism in most bacterial strains as they exhibit resistance to antibiotics by various means like restricting penetration of antibiotics into biofilms, decreasing growth rate, and expressing resistance genes. It is noted that biofilm formation allows microorganisms to survive and thrive in a hostile environment, to disperse forming new niches, and to give them significant advantages in protection against environmental fluctuations. Bacteria in biofilms display increased cell-to-cell communication while becoming less sensitive to chemical and physical stresses, and this may further complicate patient treatment outcome [46, 47].

Multidrug resistance (MDR) to three or more antimicrobial agents in different categories was observed in 68.7% of the tested isolates. This is a significant proportion that can influence external eye infected patient cure. Comparable findings are reported in studies conducted from southern and northern Ethiopia [18, 24]. It has been suggested that indiscriminate and prolonged use of a wide range of antibiotics and lack of personal hygiene might be a major factor leading to the emergence of multidrug-resistant strains.

In the current study, strains capable of forming biofilms were more frequently observed to be MDR phenotype. Simultaneous occurrence of most MDR and biofilm-forming strains showed that biofilm phenotype may play a great role in antimicrobial resistance, although the correlation in this study was not appreciable. Other studies reported that biofilm formation is higher in MDR bacteria [48, 49]. Phenotypic changes in the bacterial shape, physiological changes within cells, low diffusion of antibiotics across the biofilm matrix, elevated expression of efflux, and quorum sensing may be some of the reasons for this high MDR property. Most of the biofilm-forming Gram-positive and Gram-negative groups isolated in this investigation were found to be resistant to aminoglycosides, penicillins, fluoroquinolones, folate pathway inhibitors, chloramphenicol, and tetracycline. Similar previous studies have also shown that biofilm formation is higher in those listed categories of antimicrobials [50–52].

In this study, as biofilm-forming features of bacteria were assessed in *in vitro* setup, the result might be different from the real biofilm formed on external ocular anatomic site infection. Moreover, the absence of routinely performed and standardized antibiofilm susceptibility protocols worldwide may undermine the influence of bacterial biofilm in patient morbidity and health care setting related acquired eye infections. Anaerobic bacterial culture and *Chlamydia trachomatis* test were not included in this research due to resource constraints. In addition, the antibiofilm drug susceptibility test was not done for biofilm former bacterial isolates due to a lack of antimicrobial constituents for the agar dilution method.

## 5. Conclusion

The prevalence rate of bacterial isolates among external eye infections was high. Both Gram-positive and Gram-negative groups were responsible for external ocular infections with the most predominant isolates: CoNS followed by *Staphylococcus aureus*, *Streptococcus pneumoniae*, *Pseudomonas aeruginosa*, and *Klebsiella pneumoniae.* Increased resistance rate to ampicillin, penicillin, erythromycin, trimethoprim-sulphamethoxazole, tobramycin, and tetracycline was observed. Ciprofloxacin and gentamicin were found to have better activity against a number of isolates from external ocular infections. MDR bacterial isolate was prevalent and methicillin resistance was detected in about a fifth of staphylococci isolates. *P. aeruginosa* was the leading biofilm former followed by *K. pneumoniae* and CoNS. Almost all biofilm formers were MDR or vice versa.

## Figures and Tables

**Table 1:** Prevalence of bacterial isolate against age groups at JUMC eye clinic.

Name of bacterial isolate	Age in years					Total (*N* = 319)
	0–2 (*N* = 103)	3–16 (*N* = 48)	17–30 (*N* = 46)	31–45 (*N* = 48)	>45 (*N* = 74)	
Gram-positive bacteria						
*S. aureus*	9 (20.5)	2 (10.0)	7 (31.8)	2 (9.1)	9 (23.1)	**29 (19.7)**
CoNS^*∗*^	10 (22.7)	5 (25.0)	4 (18.2)	11 (50.0)	11 (28.2)	**41 (27.9)**
*S. pneumoniae*	4 (9.1)	1 (5.0)	2 (9.1)	1 (4.5)	5 (12.8)	**13 (8.8)**
*S. pyogenes*	1 (2.3)	3 (15.0)	1 (4.5)	0 (0.0)	0 (0.0)	**5 (3.4)**
*S. agalactiae*	1 (2.3)	1 (5.0)	0 (0.0)	0 (0.0)	3 (7.7)	**5 (3.4)**
S. viridians	2 (4.5)	0 (0.0)	0 (0.0)	0 (0.0)	1 (2.6)	**3 (2.0)**

Gram-negative bacteria						
*P. aeruginosa*	1 (2.3)	2 (10.0)	2 (9.1)	3 (13.6)	2 (5.1)	**10 (6.8)**
*K. pneumoniae*	2 (4.5)	2 (10.0)	1 (4.5)	1 (4.5)	3 (7.7)	**9 (6.1)**
*P. mirabilis*	1 (2.3)	1 (5.0)	2 (9.1)	0 (0.0)	1 (2.6)	**5 (3.4)**
*P. vulgaris*	1 (2.3)	1 (5.0)	0 (0.0)	1 (4.5)	1 (2.6)	**4 (2.7)**
*S. marcescens*	2 (4.5)	0 (0.0)	1 (4.5)	0 (0.0)	0 (0.0)	**3 (2.0)**
*Citrobacter* spp.	2 (4.5)	0 (0.0)	0 (0.0)	1 (4.5)	2 (5.1)	**5 (3.4)**
*Enterobacter* spp.	2 (4.5)	0 (0.0)	0 (0.0)	0 (0.0)	1 (2.6)	**3 (2.0)**
*E. coli*	1 (2.3)	1 (5.0)	1 (4.5)	2 (9.1)	0 (0.0)	**5 (3.4)**
*H. influenzae*	4 (9.1))	0 (0.0)	1 (4.5)	0 (0.0)	0 (0.0)	**5 (3.4)**
*N. meningitidis*	1 (2.3)	1 (5.0)	0 (0.0)	0 (0.0)	0 (0.0)	**2 (1.4)**
Total	**44 (29.9)**	**20 (13.6)**	**22 (15.0)**	**22 (15.0)**	**39 (26.5)**	**147 (100)**

^*∗*^Coagulase-negative staphylococci.

**Table 2:** Distribution of bacteria isolates against the different clinical features of external ocular infections at JUMC eye clinic.

Name of bacterial isolate	Types of diagnosis					Total (*N* = 319)
	Conjunctivitis (*N* = 165)	Blepharitis (*N* = 52)	Blepharoconjunctivitis (*N* = 74)	Dacryocystitis (*N* = 13)	Others (*N* = 15)	
Gram-positive bacteria						
*S. aureus*	15 (20.0)	8 (25.0)	1 (3.7)	2 (25.0)	3 (60)	**29 (19.7)**
CoNS^*∗*^	16 (21.3)	10 (31.2)	10 (37.0)	3 (37.5)	2 (40)	**41 (27.9)**
*S. pneumoniae*	9 (12.0)	2 (6.2)	0 (0.0)	2 (25.0)	0 (0.0)	**13 (8.8)**
*S. pyogenes*	4 (5.3)	0 (0.0)	1 (3.7)	0 (0.0)	0 (0.0)	**5 (3.4)**
*S. agalactiae*	2 (2.7)	2 (6.2)	1 (3.7)	0 (0.0)	0 (0.0)	**5 (3.4)**
*S. viridians* ^#^	2 (2.7)	1 (3.1)	0 (0.0)	0 (0.0)	0 (0.0)	**3 (2.0)**

Gram-negative bacteria						
*P. aeruginosa*	5 (6.7)	3 (9.4)	2 (7.4)	0 (0.0)	0 (0.0)	**10 (6.8)**
*K. pneumoniae*	4 (5.3)	2 (6.2)	2 (7.4)	1 (12.5)	0 (0.0)	**9 (6.1)**
*P. mirabilis*	3 (4.0)	1 (3.1)	1 (3.7)	0 (0.0)	0 (0.0)	**5 (3.4)**
*P. vulgaris*	2 (2.7)	0 (0.0)	2 (7.4)	0 (0.0)	0 (0.0)	**4 (2.7)**
*S. marcescens*	3 (4.0)	0 (0.0)	0 (0.0)	0 (0.0)	0 (0.0)	**3 (2.0)**
*Citrobacter* spp.	3 (4.0)	1 (3.1)	1 (3.7)	0 (0.0)	0 (0.0)	**5 (3.4)**
*Enterobacter* spp.	2 (2.7)	0 (0.0)	1 (3.7)	0 (0.0)	0 (0.0)	**3 (2.0)**
*E. coli*	2 (2.7)	2 (6.2)	1 (3.7)	0 (0.0)	0 (0.0)	**5 (3.4)**
*H. influenzae*	2 (2.7)	0 (0.0)	3 (11.1)	0 (0.0)	0 (0.0)	**5 (3.4)**
*N. meningitidis*	1 (1.3)	0 (0.0)	1 (3.7)	0 (0.0)	0 (0.0)	**2 (1.4)**
Total	**75 (51.0)**	**32 (21.8)**	**27 (18.4)**	**8 (5.4)**	**5 (3.4)**	**147 (100)**

^*∗*^Coagulase-negative staphylococcus; ^#^*Viridans streptococci*.

**Table 3:** Antimicrobial susceptibility patterns of Gram-positive isolates from external eye infection at JUMC ophthalmic clinic.

Bacterial isolate	Antimicrobial agents tested													
	Total	Pattern	AMC no. (%)	AMP no. (%)	CIP no. (%)	AK no. (%)	C no. (%)	CLN no. (%)	TE no. (%)	SXT no. (%)	ERY no. (%)	CN no. (%)	FOX no. (%)	P no. (%)
*S. aureus*	29	S	22 (75.9)	5 (17.2)	26 (89.7)	20 (68.9)	16 (55.2)	24 (82.8)	7 (24.1)	9 (31.0)	5 (17.2)	21 (72.4)	25 (86.2)	4 (13.8)
		R	7 (24.1)	24 (82.8)	3 (10.3)	9 (31.0)	13 (44.8)	5 (17.2)	22 (75.9)	20 (69.0)	24 (82.8)	8 (27.6)	4 (13.8)	25 (86.2)

CoNS	41	S	37 (90.2)	7 (17.1)	32 (78.0)	30 (73.2)	23 (56.1)	33 (80.5)	12 (29.3)	13 (31.7)	11 (26.8)	32 (78.0)	29 (70.7)	7 (17.1)
		R	4 (9.8)	34 (82.9)	9 (22.0)	11 (26.8)	18 (43.9)	8 (19.5)	29 (70.7)	28 (68.3)	30 (73.2)	9 (22.0)	12 (29.3)	34 (82.9)

*S. pneumoniae*	13	S	13 (100)	5 (38.5)	11 (84.6)	10 (76.9)	10 (76.9)	8 (61.5)	5 (38.5)	4 (30.8)	7 (53.8)	10 (76.9)	NT	4 (30.8)
		R	0 (0.0)	8 (61.5)	2 (15.4)	3 (23.1)	3 (23.1)	5 (38.5)	8 (61.5)	9 (69.2)	6 (46.2)	3 (23.1)	NT	9 (69.2)

*S. pyogenes*	5	S	5 (100)	2 (40.0)	4 (80.0)	4 (80.0)	4 (80.0)	4 (80.0)	2 (40.0)	2 (40.0)	3 (60.0)	4 (80.0)	NT	3 (60.0)
		R	0 (0.0)	3 (60.0)	1 (20.0)	1 (20.0)	1 (20.0)	1 (20.0)	3 (60.0)	3 (60.0)	2 (40.0)	1 (20.0)	NT	2 (40.0)

*S. agalactiae*	5	S	5 (100)	2 (40.0)	5 (100)	4 (80.0)	4 (80.0)	5 (100)	2 (40.0)	3 (60.0)	4 (80.0)	4 (80.0)	NT	3 (60.0)
		R	0 (0.0)	3 (60.0)	0 (0.0)	1 (20.0)	1 (20.0)	0 (0.0)	3 (60.0)	2 (40.0)	1 (20.0)	1 (20.0)	NT	2 (40.0)

*S. viridians* ^#^	3	S	3 (100)	2 (66.7)	3 (100)	3 (100)	3 (100)	3 (100)	2 (66.7)	1 (33.3)	2 (66.7)	3 (100)	NT	1 (33.3)
		R	0 (0.0)	1 (33.3)	0 (0.0)	0 (0.0)	0 (0.0)	0 (0.0)	1 (33.3)	2 (66.7)	1 (33.3)	0 (0.0)	NT	2 (66.7)

CoNS: coagulase-negative staphylococci; S: susceptible; R: resistance; AMC: amoxicillin-clavulanic acid; AMP: ampicillin; CIP: ciprofloxacin; AK: amikacin; C: chloramphenicol; CLN: clindamycin; TE: tetracycline; SXT: trimethoprim-sulphamethoxazole; ERY: erythromycin; CN: gentamicin; FOX: cefoxitin; P: penicillin G; no.: number; NT: not tested. ^#^*Viridans streptococci*. A few Intermediate susceptible isolates were included in the susceptible category.

**Table 4:** Antimicrobial susceptibility patterns of Gram-negative isolates from an external eye infection at JUMC ophthalmic clinic.

Bacterial isolate	Antimicrobial agents tested												
	Total	Pattern	AMC no. (%)	AMP no. (%)	CIP no. (%)	CRO no. (%)	C no. (%)	CAZ no. (%)	TE no. (%)	SXT no. (%)	CN no. (%)	TOB no. (%)	AK no. (%)
*P. aeruginosa*	10	S	8 (80.0)	5 (50.0)	9 (90.0)	8 (80.0)	5 (50.0)	1 (10.0)	2 (20.0)	3 (30.0)	8 (80.0)	3 (30.0)	8 (80.0)
		R	2 (20.0)	5 (50.0)	1 (10.0)	2 (20.0)	5 (50.0)	9 (90.0)	8 (80.0)	7 (70.0)	2 (20.0)	7 (70.0)	2 (20.0)

*K. pneumoniae*	9	S	8 (88.9)	6 (66.7)	9 (100)	9 (100)	8 (88.9)	6 (66.7)	4 (44.4)	4 (44.4)	8 (88.9)	4 (44.4)	8 (88.9)
		R	1 (11.1)	3 (33.3)	0 (0.0)	0 (0.0)	1 (11.1)	3 (33.3)	5 (55.6)	5 (55.6)	1 (11.1)	5 (55.6)	1 (11.1)

*P. mirabilis*	5	S	5 (100)	4 (80.0)	5 (100)	4 (80.0)	4 (80.0)	4 (80.0)	3 (60.0)	3 (60.0)	5 (100)	2 (40.0)	5 (100)
		R	0 (0.0)	1 (20.0)	0 (0.0)	1 (20.0)	1 (20.0)	1 (20.0)	2 (40.0)	2 (40.0)	0 (0.0)	3 (60.0)	0 (0.0)

*P. vulgaris*	4	S	4 (100)	3 (75.0)	4 (100)	4 (100)	4 (100)	3 (75.0)	3 (75.0)	3 (75.0)	3 (75.5)	1 (25.0)	3 (75.0)
		R	0 (0.0)	1 (25.0)	0 (0.0)	0 (0.0)	0 (0.0)	1 (25.0)	1 (25.0)	1 (25.0)	1 (25.0)	3 (75.0)	1 (25.0)

*S. marcescens*	3	S	3 (100)	1 (66.7)	3 (100)	3 (100)	3 (100)	3 (100)	2 (66.7)	0 (0.0)	3 (100)	1 (33.3)	2 (66.7)
		R	0 (0.0)	1 (33.3)	0 (0.0)	0 (0.0)	0 (0.0)	0 (0.0)	2 (33.3)	3 (100)	0 (0.0)	2 (66.7)	1 (33.3)

*Citrobacter* spp.	5	S	4 (80.0)	4 (80.0)	5 (100)	5 (100)	4 (80.0)	4 (80.0)	3 (60.0)	5 (100)	4 (80.0)	3 (60.0)	4 (80.0)
		R	1 (20.0)	1 (20.0)	0 (0.0)	0 (0.0)	1 (20.0)	1 (20.0)	2 (40.0)	0 (0.0)	1 (20.0)	2 (40.0)	1 (20.0)

*Enterobacter* spp.	3	S	3 (100)	2 (66.7)	3 (100)	3 (100)	3 (100)	3 (100)	1 (33.3)	3 (100)	2 (66.7)	1 (33.3)	2 (66.7)
		R	0 (0.0)	1 (33.3)	0 (0.0)	0 (0.0)	0 (0.0)	0 (0.0)	2 (66.7)	0 (0.0)	1 (33.3)	2 (66.7)	1 (33.3)

*E. coli*	5	S	4 (80)	3 (60.0)	5 (100)	5 (100)	3 (60.0)	4 (80.0)	1 (20.0)	4 (80.0)	5 (100)	2 (40.0)	4 (80.0)
		R	1 (20.0)	2 (40.0)	0 (0.0)	0 (0.0)	2 (40.0)	1 (20.0)	4 (80.0)	2 (40.0)	0 (0.0)	3 (60.0)	1 (20.0)

*H. influenzae*	5	S	4 (80.0)	3 (60.0)	5 (100)	5 (100)	4 (80.0)	4 (80.0)	3 (60.0)	3 (60.0)	4 (80.0)	NT	NT
		R	1 (20.0)	2 (40.0)	0 (0.0)	0 (0.0)	1 (20.0)	1 (20.0)	2 (40.0)	2 (40.0)	1 (20.0)	NT	NT

*N. meningitidis*	2	S	NT	NT	2 (100)	2 (100)	1 (50.0)	NT	NT	1 (50.0)	NT	NT	NT
		R	NT	NT	0 (0.0)	0 (0.0)	1 (50.0)	NT	NT	1 (50.0)	NT	NT	NT

S: susceptible; R: resistance; AMC: amoxicillin-clavulanic acid; AMP: ampicillin; CIP: ciprofloxacin; AK: amikacin; C: chloramphenicol; TE : tetracycline; SXT: trimethoprim-sulphamethoxazole; CN: gentamicin; CRO: ceftriaxone; CAZ: ceftazidime; TOB: tobramycin; no.: number; NT: not tested. A few intermediate susceptible isolates were included in the susceptible category.

**Table 5:** MDR pattern of bacteria isolated from external ocular infected patients at JUMC eye clinic.

Bacterial isolates	Total	Antibiotic resistance pattern					
		R0	R1	R2	R3	R4	≥R5
*S. aureus*	29	0 (0.0)	0 (0.0)	4 (13.8)	5 (17.2)	7 (24.1)	13 (44.8)
CoNS	41	0 (0.0)	2 (4.9)	5 (12.2)	8 (19.5)	10 (24.4)	16 (39.0)
*S. pneumoniae*	13	0 (0.0)	1 (7.7)	3 (23.1)	3 (23.1)	6 (46.2)	0 (0.0)
*S. pyogenes*	5	0 (0.0)	1 (20.0)	0 (0.0)	2 (40.0)	1 (20.0)	1 (20.0)
*S. agalactiae*	5	1 (20.0)	1 (20.0)	1 (20.0)	1 (20.0)	0 (0.0)	1 (20.0)
S. viridians	3	0 (0.0)	2 (66.7)	0 (0.0)	1 (33.3)	0 (0.0)	0 (0.0)
*P. aeruginosa*	10	0 (0.0)	0 (0.0)	0 (0.0)	3 (30.0)	2 (20.0)	5 (50.0)
*K. pneumoniae*	9	1 (11.1)	1 (11.1)	1 (11.1)	4 (44.4)	2 (22.2)	0 (0.0)
*P. mirabilis*	5	0 (0.0)	2 (40.0)	2 (40.0)	0 (0.0)	0 (0.0)	1 (20.0)
*P. vulgaris*	4	0 (0.0)	2 (50.0)	1 (25.0)	1 (25.0)	0 (0.0)	0 (0.0)
*S. marcescens*	3	0 (0.0)	0 (0.0)	2 (66.7)	1 (33.3)	0 (0.0)	0 (0.0)
*Citrobacter* spp.	5	1 (20.0)	2 (40.0)	0 (0.0)	2 (40.0)	0 (0.0)	0 (0.0)
*Enterobacter* spp.	3	0 (0.0)	1 (33.3)	1 (33.3)	1 (33.3)	0 (0.0)	0 (0.0)
*E. coli*	5	0 (0.0)	1 (20.0)	1 (20.0)	1 (20.0)	1 (20.0)	1 (20.0)
*H. influenzae*	5	1 (20.0)	2 (40.0)	1 (20.0)	1 (20.0)	0 (0.0)	0 (0.0)
*N. meningitidis*	2	0 (0.0)	2 (100)	0 (0.0)	0 (0.0)	0 (0.0)	0 (0.0)
Total	**147**	**4 (2.7)**	**20 (13.6)**	**22 (15.0)**	**34 (23.1)**	**29 (19.7)**	**38 (25.9)**

CoNS: coagulase-negative staphylococci; R*o*: bacterial isolate susceptible to all antimicrobial agents tested; R1: bacterial isolate resistance to 1 antimicrobial agent; R2: bacterial isolate resistance to 2 antimicrobial agents of different classes; R3: bacterial isolate resistance to 3 antimicrobial agents of different classes; R4: bacterial isolate resistance to 4 antimicrobial agents of different classes; ≥R5: bacterial isolate resistance to 5 or more antimicrobial agents of different classes.

**Table 6:** Biofilm forming capability and adherence classification of bacteria from external ocular infected patients at JUMC ophthalmic clinic.

Bacterial isolates	Biofilm formation classification				
	Total^*∗*^	Strong	Moderate	Weak	Nonadherent
*S. aureus*	29	9 (31.0)	10 (34.5)	2 (6.9)	8 (27.6)
CoNS	41	14 (34.1)	13 (31.7)	4 (9.8)	10 (24.4)
*S. pyogenes*	5	0 (0.0)	1 (20.0)	1 (20.0)	3 (60.0)
*S. agalactiae*	5	0 (0.0)	1 (20.0)	1 (20.0)	3 (60.0)
S. viridians	3	0 (0.0)	0 (0.0)	0 (0.0)	3 (100)
*P. aeruginosa*	10	4 (40.0)	3 (30.0)	1 (10.0)	2 (20.0)
*K. pneumoniae*	9	2 (22.2)	3 (33.3)	2 (22.2)	2 (22.2)
*P. mirabilis*	5	1 (20.0)	1 (20.0)	1 (20.0)	2 (40.0)
*P. vulgaris*	4	0 (0.0)	0 (0.0)	2 (50.0)	2 (50.0)
*S. marcescens*	3	0 (0.0)	1 (33.3)	0 (0.0)	2 (66.7)
*Citrobacter* spp.	5	0 (0.0)	3 (60.0)	0 (0.0)	2 (40.0)
*Enterobacter* spp.	3	0 (0.0)	1 (33.3)	0 (0.0)	2 (66.7)
*E. coli*	5	1 (20.0)	2 (40.0)	0 (0.0)	2 (40.0)
Total	**127**	**31 (24.4)**	**39 (30.7)**	**14 (11.0)**	**43 (33.9)**

^*∗*^ *S. pneumoniae* (*n* = 13), *H. influenzae* (*n* = 5), and *N. meningitidis* (*n* = 2) altogether, their biofilm formation capability was not assessed for they are delicate and fastidious by nature.

## Data Availability

All the data sets used to support the findings of this study are available from the corresponding author upon request.
